# Supplementary material for: Development and Validation of Confirmatory Foot-and-Mouth Disease Virus Antibody ELISAs to Identify Infected Animals in Vaccinated Populations
Source: Viruses. 2021 May 15;13(5):914. doi: 10.3390/v13050914 (PMC8156621; doi:10.3390/v13050914)
Supplement: Supplementary file 1 [file viruses-13-00914-s001.zip › viruses-1180446-SI/viruses-1180446-SI.pdf]

Supplementary Information

Supplementary Table S1. Assessment of the diagnostic agreement between either the PrioCHECK® or IDvet FMDV NS ELISAs and the in-house ELISA tests for detecting infection and/or carrier status in unvaccinated, vaccinated, bovine NSP panel, and cattle field sera. κ = kappa statistic; PPA = positive agreement (%); NPA = negative agreement (%).

	**Prionics**			**IDvet Long**			**IDvet Short**		
	**κ**	**PPA**	**NPA**	**κ**	**PPA**	**NPA**	**κ**	**PPA**	**NPA**
Unavaccinated Infected Recovered									
2B	0.87	88.68	99.19	0.82	88.68	99.19	0.85	90.38	99.19
3B	0.82	88.68	98.37	0.85	88.68	98.37	0.86	90.38	98.38
3ABC	0.87	88.68	99.19	0.82	88.68	99.19	0.87	90.38	99.19
3D	0.81	86.79	97.26	0.82	86.79	97.26	0.85	90.38	97.36
3CD	0.81	86.79	98.56	0.83	86.79	98.58	0.85	88.46	98.58
2C	0.17	75.47	76.85	0.12	73.58	76.75	0.13	75.00	76.77
Unvaccinated Carrier									
2B	0.86	78.57	99.19	0.87	78.57	99.19	0.88	81.48	99.19
3B	0.88	78.57	98.37	0.88	78.57	98.37	0.86	81.48	98.38
3ABC	0.86	78.57	99.19	0.87	78.57	99.19	0.88	81.48	99.19
3D	0.84	75.00	97.26	0.85	75.00	97.26	0.86	81.48	97.36
3CD	0.86	75.00	98.58	0.87	75.00	98.58	0.88	77.78	98.58
2C	0.06	57.14	76.85	0.06	53.57	76.75	0.07	55.55	76.77
Vaccinated Infected Recovered									
2B	0.74	67.58	98.85	0.77	73.30	98.11	0.76	72.86	98.57
3B	0.85	84.02	98.18	0.84	85.86	97.08	0.82	85.43	97.62
3ABC	0.77	69.86	99.14	0.79	75.92	98.49	0.79	74.37	98.76
3D	0.67	70.32	95.31	0.66	72.25	94.34	0.65	71.86	94.78
3CD	0.66	63.01	97.22	0.66	64.92	96.42	0.65	65.33	96.96
2C	0.18	47.03	77.01	0.19	49.21	76.72	0.22	49.25	76.92
Vaccinated Carrier									
2B	0.77	71.72	98.90	0.76	73.38	98.71	0.77	73.68	98.32
3B	0.81	79.31	98.20	0.81	82.73	98.21	0.83	82.71	97.73
3ABC	0.81	75.17	99.20	0.81	76.26	98.91	0.80	78.19	98.71
3D	0.64	62.76	97.00	0.63	64.03	96.82	0.64	64.66	96.54
3CD	0.64	57.93	98.30	0.62	58.99	98.11	0.64	58.65	97.73
2C	0.17	50.34	76.80	0.16	50.36	76.64	0.17	50.37	76.48
Panel									
2B	0.77	79.49	98.99	0.76	79.49	98.99	0.76	81.08	98.89
3B	0.70	76.92	98.18	0.71	79.49	98.28	0.71	81.08	98.18
3ABC	0.68	64.10	99.09	0.72	66.67	99.19	0.70	70.27	99.19
3D	0.50	61.54	97.06	0.53	64.10	97.16	0.52	67.57	97.17
3CD	0.60	61.54	98.38	0.60	64.10	98.48	0.61	64.86	98.38
2C	0.10	64.10	76.82	0.08	56.41	76.52	0.09	56.76	76.46
Field									
2B	0.93	93.21	99.05	0.93	92.73	99.19	0.92	94.37	98.99
3B	0.92	95.06	98.10	0.92	93.94	98.37	0.91	96.25	98.28
3ABC	0.94	93.83	98.95	0.94	92.73	99.19	0.92	95.62	99.19
3D	0.74	73.46	96.96	0.74	72.73	97.26	0.73	75.00	97.27
3CD	0.75	69.13	98.29	0.74	68.48	98.58	0.74	70.00	98.48
2C	0.37	80.25	75.78	0.37	78.79	76.75	0.37	80.62	76.77

Supplementary Table S2. Assessment of the diagnostic performance for a range of ±0.1 cut-off points of each of the NSP tests, for the detection of infection and/or carrier status in. unvaccinated, vaccinated, published NSP panel, and known clinically infected field sera from cattle. Se = sensitivity (%); Sp = specificity (%), LR+ = likelihood ratio test for positive results; LR- = likelihood ratio test for negative results.

	**Test**	**Cut-Off**	**Se**	**Sp**	**Classified**	**LR+**	**LR-**
Unvaccinated Infected Recovered	Prionics	40	100.00	94.85	95.09	19.43	0.00
		45	100.00	98.08	98.17	52.16	0.00
		50	100.00	99.39	99.42	165.17	0.00
		55	100.00	99.60	99.61	247.75	0.00
		60	100.00	99.80	99.81	495.51	0.00
	IDvet Long	40	100.00	98.59	98.65	70.79	0.00
		45	100.00	98.69	98.75	76.23	0.00
		50	100.00	99.29	99.33	141.57	0.00
		55	100.00	99.50	99.52	198.20	0.00
		60	100.00	99.70	99.71	330.33	0.00
	IDvet Short	40	100.00	98.99	99.04	99.10	0.00
		45	100.00	99.19	99.23	123.87	0.00
		50	100.00	99.39	99.42	165.17	0.00
		55	100.00	99.50	99.52	198.20	0.00
		60	100.00	99.60	99.61	247.75	0.00
	2B	0.4	100.00	96.97	97.11	33.03	0
		0.45	100.00	98.08	98.17	52.16	0
		0.5	100.00	99.09	99.13	110.11	0
		0.55	95.74	99.60	99.42	237.21	0.04
		0.6	93.62	99.70	99.42	309.25	0.06
	3B	0.4	100.00	84.66	85.36	6.52	0.00
		0.45	100.00	92.53	92.87	13.39	0.00
		0.5	100.00	98.39	98.46	61.94	0.00
		0.55	97.87	98.79	98.75	80.83	0.02
		0.6	95.74	98.89	98.75	86.26	0.04
	3ABC	0.4	100.00	96.27	96.44	26.78	0.00
		0.45	100.00	98.18	98.27	55.06	0.00
		0.5	100.00	99.09	99.13	110.11	0.00
		0.55	93.62	99.19	98.94	115.97	0.06
		0.6	89.36	99.29	98.84	126.51	0.11
	3D	0.4	97.87	96.77	96.82	30.31	0.02
		0.45	97.87	96.97	97.01	32.33	0.02
		0.5	97.87	97.17	97.21	34.64	0.02
		0.55	97.87	97.88	97.88	46.19	0.02
		0.6	91.49	98.08	97.78	47.72	0.09
	3CD	0.4	97.87	98.08	98.07	51.05	0.02
		0.45	97.87	98.28	98.27	57.05	0.02
		0.5	97.87	98.49	98.46	64.66	0.02
		0.55	93.62	98.89	98.65	84.34	0.06
		0.6	91.49	99.29	98.94	129.52	0.09
	2C	0.4	87.23	65.29	66.28	2.51	0.20
		0.45	82.98	71.95	72.45	2.96	0.24
		0.5	82.98	76.69	76.97	3.56	0.22
		0.55	78.72	80.52	80.44	4.04	0.26
		0.6	76.60	83.96	83.62	4.77	0.28
Unvaccinated Carrier	Prionics	40	100.00	94.85	94.97	19.43	0.00
		45	100.00	98.08	98.12	52.16	0.00
		50	100.00	99.39	99.41	165.17	0.00
		55	100.00	99.60	99.61	247.75	0.00
		60	100.00	99.80	99.80	495.51	0.00

	IDvet Long	40	100.00	98.59	98.62	70.79	0.00
		45	100.00	98.69	98.72	76.23	0.00
		50	100.00	99.29	99.31	141.57	0.00
		55	100.00	99.50	99.51	198.20	0.00
		60	100.00	99.70	99.70	330.33	0.00
	IDvet Short	40	100.00	98.99	99.01	99.10	0.00
		45	100.00	99.19	99.21	123.87	0.00
		50	100.00	99.39	99.41	165.17	0.00
		55	100.00	99.50	99.51	198.20	0.00
		60	100.00	99.60	99.61	247.75	0.00
	2B	0.4	100.00	96.97	97.04	33.03	0.00
		0.45	100.00	98.59	98.62	70.79	0.00
		0.5	100.00	99.09	99.11	110.11	0.00
		0.55	90.91	99.60	99.41	225.23	0.09
		0.6	86.36	99.70	99.41	285.29	0.14
	3B	0.4	100.00	84.66	85.00	6.52	0.00
		0.45	100.00	92.53	92.69	13.39	0.00
		0.5	100.00	98.39	98.42	61.94	0.00
		0.55	100.00	98.79	98.82	82.58	0.00
		0.6	95.45	98.79	98.72	78.83	0.05
	3ABC	0.4	100.00	96.27	96.35	26.78	0.00
		0.45	100.00	98.18	98.22	55.06	0.00
		0.5	100.00	99.09	99.11	110.11	0.00
		0.55	90.91	99.19	99.01	112.61	0.09
		0.6	86.36	99.29	99.01	122.27	0.14
	3D	0.4	95.45	96.77	96.74	29.56	0.05
		0.45	95.45	96.97	96.94	31.53	0.05
		0.5	95.45	97.17	97.14	33.78	0.05
		0.55	95.45	97.88	97.83	45.05	0.05
		0.6	86.36	97.98	97.73	42.79	0.14
	3CD	0.4	95.45	98.08	98.03	49.79	0.05
		0.45	95.45	98.28	98.22	55.64	0.05
		0.5	95.45	98.49	98.42	63.06	0.05
		0.55	90.91	98.89	98.72	81.90	0.09
		0.6	90.91	99.19	99.01	112.61	0.09
	2C	0.4	77.27	65.29	65.55	2.23	0.35
		0.45	68.18	71.95	71.87	2.43	0.44
		0.5	68.18	76.69	76.51	2.93	0.41
		0.55	59.09	80.52	80.06	3.03	0.51
		0.6	59.09	83.96	83.42	3.68	0.49
Vaccinated Infected Recovered	Prionics	40	85.83	94.83	93.05	16.59	0.15
		45	84.21	98.11	95.37	44.54	0.16
		50	82.59	99.40	96.09	138.34	0.18
		55	82.19	99.80	96.33	412.99	0.18
		60	80.16	99.90	96.01	805.63	0.20
	IDvet Long	40	79.76	98.51	94.81	53.44	0.21
		45	76.92	98.61	94.33	55.22	0.23
		50	74.90	99.20	94.41	94.09	0.25
		55	72.87	99.40	94.17	122.06	0.27
		60	70.04	99.60	93.77	175.98	0.30
	IDvet Short	40	82.59	98.81	95.61	69.17	0.18
		45	80.16	99.10	95.37	89.51	0.20
		50	77.73	99.30	95.05	111.60	0.22
		55	74.90	99.40	94.57	125.46	0.25
		60	72.47	99.60	94.25	182.08	0.28
	2B	0.4	67.21	97.01	91.13	22.51	0.34
		0.45	63.56	98.01	91.21	31.94	0.37
		0.5	61.54	99.10	91.69	68.72	0.39
		0.55	55.87	99.40	90.81	93.58	0.44
		0.6	48.58	99.60	89.54	122.06	0.52
	3B	0.4	80.97	84.68	83.95	5.28	0.22
		0.45	77.73	92.54	89.62	10.42	0.24
		0.5	72.47	98.41	93.29	45.52	0.28
		0.55	70.45	98.81	93.21	59.00	0.30
		0.6	65.18	98.91	92.25	59.55	0.35

	3ABC	0.4	65.18	96.22	90.10	17.24	0.36
		0.45	62.75	98.21	91.21	35.04	0.38
		0.5	61.54	99.00	91.61	61.85	0.39
		0.55	57.09	99.20	90.89	71.71	0.43
		0.6	51.42	99.20	89.78	64.59	0.49
	3D	0.4	70.04	96.22	91.05	18.52	0.31
		0.45	68.83	96.52	91.05	19.76	0.32
		0.5	66.80	96.62	90.73	19.75	0.34
		0.55	62.75	97.51	90.65	25.23	0.38
		0.6	57.49	97.61	89.70	24.07	0.44
	3CD	0.4	61.54	97.51	90.42	24.74	0.39
		0.45	59.92	97.61	90.18	25.09	0.41
		0.5	57.89	98.11	90.18	30.62	0.43
		0.55	52.23	98.51	89.38	34.99	0.49
		0.6	49.80	98.81	89.14	41.71	0.51
	2C	0.4	55.06	65.47	63.42	1.59	0.69
		0.45	50.20	72.04	67.73	1.80	0.69
		0.5	44.13	76.92	70.45	1.91	0.73
		0.55	35.22	80.60	71.65	1.82	0.80
		0.6	30.36	83.98	73.40	1.90	0.83
Vaccinated Carrier	Prionics	40	92.86	94.85	94.59	18.04	0.08
		45	90.91	98.08	97.12	47.42	0.09
		50	90.26	99.39	98.17	149.08	0.10
		55	90.26	99.80	98.52	447.25	0.10
		60	88.31	99.90	98.34	875.12	0.12
	IDvet Long	40	90.26	98.59	97.47	63.89	0.10
		45	88.96	98.79	97.47	73.47	0.11
		50	86.36	99.29	97.55	122.27	0.14
		55	83.77	99.60	97.47	207.53	0.16
		60	80.52	99.80	97.21	398.98	0.20
	IDvet Short	40	88.96	98.99	97.64	88.16	0.11
		45	87.01	99.29	97.64	123.19	0.13
		50	83.77	99.50	97.38	166.03	0.16
		55	81.82	99.60	97.21	202.70	0.18
		60	77.92	99.70	96.77	257.40	0.22
	2B	0.4	75.32	97.07	94.15	25.74	0.25
		0.45	72.08	98.08	94.59	37.59	0.28
		0.5	70.13	99.19	95.28	86.87	0.30
		0.55	66.23	99.50	95.02	131.28	0.34
		0.6	57.79	99.70	94.06	190.91	0.42
	3B	0.4	83.77	84.66	84.54	5.46	0.19
		0.45	81.82	92.53	91.09	10.96	0.20
		0.5	75.97	98.39	95.37	47.06	0.24
		0.55	74.03	98.79	95.46	61.13	0.26
		0.6	66.23	98.89	94.50	59.67	0.34
	3ABC	0.4	73.38	96.27	93.19	19.65	0.28
		0.45	71.43	98.18	94.59	39.33	0.29
		0.5	70.78	99.09	95.28	77.94	0.29
		0.55	64.94	99.29	94.67	91.93	0.35
		0.6	59.09	99.29	93.89	83.66	0.41
	3D	0.4	63.64	96.77	92.31	19.71	0.38
		0.45	62.34	97.07	92.40	21.30	0.39
		0.5	61.04	97.17	92.31	21.60	0.40
		0.55	57.79	97.98	92.58	28.64	0.43
		0.6	53.90	98.08	92.14	28.11	0.47
	3CD	0.4	59.74	98.08	92.93	31.16	0.41
		0.45	59.09	98.28	93.01	34.45	0.42
		0.5	56.49	98.59	92.93	39.99	0.44
		0.55	49.35	98.99	92.31	48.91	0.51
		0.6	46.10	99.19	92.05	57.11	0.54
	2C	0.4	63.64	65.29	65.07	1.83	0.56
		0.45	57.14	71.95	69.96	2.04	0.60
		0.5	49.35	76.89	73.19	2.14	0.66
		0.55	40.26	80.52	75.11	2.07	0.74
		0.6	35.71	83.96	77.47	2.23	0.77

Panel	Prionics	40	94.44	94.85	94.84	18.35	0.06
		45	94.44	98.08	97.96	49.26	0.06
		50	91.67	99.39	99.12	151.40	0.08
		55	91.67	99.60	99.32	227.10	0.08
		60	86.11	99.80	99.32	426.69	0.14
	IDvet Long	40	100.00	98.59	98.64	70.79	0.00
		45	100.00	98.79	98.83	82.58	0.00
		50	91.67	99.29	99.03	129.77	0.08
		55	91.67	99.50	99.22	181.68	0.08
		60	86.11	99.80	99.32	426.69	0.14
	IDvet Short	40	94.44	98.99	98.83	93.59	0.06
		45	94.44	99.19	99.03	116.99	0.06
		50	91.67	99.50	99.22	181.68	0.08
		55	88.89	99.50	99.12	176.18	0.11
		60	86.11	99.60	99.12	213.34	0.14
	2B	0.4	97.22	96.97	96.98	32.12	0.03
		0.45	94.44	98.08	97.96	49.26	0.06
		0.5	94.44	99.09	98.93	103.99	0.06
		0.55	75.00	99.50	98.64	148.65	0.25
		0.6	69.44	99.70	98.64	229.40	0.31
	3B	0.4	94.44	84.66	85.00	6.16	0.07
		0.45	94.44	92.53	92.60	12.65	0.06
		0.5	88.89	98.39	98.05	55.06	0.11
		0.55	88.89	98.79	98.44	73.41	0.11
		0.6	86.11	98.79	98.34	71.11	0.14
	3ABC	0.4	86.11	96.27	95.91	23.06	0.14
		0.45	75.00	98.18	97.37	41.29	0.25
		0.5	75.00	99.19	98.34	92.91	0.25
		0.55	69.44	99.29	98.25	98.31	0.31
		0.6	66.67	99.29	98.15	94.38	0.34
	3D	0.4	80.56	96.87	96.30	25.75	0.20
		0.45	72.22	96.97	96.11	23.86	0.29
		0.5	72.22	97.17	96.30	25.56	0.29
		0.55	69.44	97.88	96.88	32.77	0.31
		0.6	66.67	97.98	96.88	33.03	0.34
	3CD	0.4	75.00	98.08	97.27	39.12	0.25
		0.45	75.00	98.28	97.47	43.72	0.25
		0.5	72.22	98.49	97.57	47.71	0.28
		0.55	58.33	98.89	97.47	52.55	0.42
		0.6	50.00	99.29	97.57	70.79	0.50
	2C	0.4	72.22	65.29	65.53	2.08	0.43
		0.45	72.22	71.95	71.96	2.57	0.39
		0.5	69.44	76.69	76.44	2.98	0.40
		0.55	63.89	80.52	79.94	3.28	0.45
		0.6	61.11	83.96	83.15	3.81	0.46
Field	Prionics	40	97.48	94.85	95.22	18.94	0.03
		45	97.48	98.08	98.00	50.85	0.03
		50	96.86	99.39	99.04	159.97	0.03
		55	96.86	99.60	99.22	239.96	0.03
		60	96.86	99.80	99.39	479.93	0.03
	IDvet Long	40	100.00	98.59	98.78	70.79	0.00
		45	100.00	98.69	98.87	76.23	0.00
		50	100.00	99.29	99.39	141.57	0.00
		55	100.00	99.50	99.57	198.20	0.00
		60	100.00	99.70	99.74	330.33	0.00
	IDvet Short	40	98.11	98.99	98.87	97.23	0.02
		45	98.11	99.29	99.13	138.90	0.02
		50	97.48	99.39	99.13	161.01	0.03
		55	97.48	99.50	99.22	193.21	0.03
		60	96.86	99.60	99.22	239.96	0.03
	2B	0.4	98.11	96.97	97.13	32.41	0.02
		0.45	96.23	98.08	97.83	50.19	0.04
		0.5	96.23	99.09	98.70	105.96	0.04
		0.55	93.71	99.50	98.70	185.74	0.06
		0.6	91.82	99.70	98.61	303.32	0.08

3B	0.4	98.11	84.66	86.52	6.40	0.02
	0.45	97.48	92.53	93.22	13.06	0.03
	0.5	97.48	98.39	98.26	60.38	0.03
	0.55	96.86	98.69	98.43	73.83	0.03
	0.6	94.97	98.89	98.35	85.56	0.05
3ABC	0.4	96.86	96.27	96.35	25.94	0.03
	0.45	96.23	98.18	97.91	52.98	0.04
	0.5	96.23	99.09	98.70	105.96	0.04
	0.55	96.23	99.19	98.78	119.20	0.04
	0.6	96.23	99.29	98.87	136.23	0.04
3D	0.4	77.36	96.77	94.09	23.96	0.23
	0.45	76.73	97.07	94.26	26.22	0.24
	0.5	75.47	97.17	94.17	26.71	0.25
	0.55	69.18	97.88	93.91	32.65	0.31
	0.6	63.52	98.08	93.30	33.13	0.37
3CD	0.4	76.73	98.08	95.13	40.02	0.24
	0.45	73.58	98.39	94.96	45.58	0.27
	0.5	71.07	98.49	94.70	46.95	0.29
	0.55	58.49	98.99	93.39	57.96	0.42
	0.6	50.31	99.29	92.52	71.23	0.50
2C	0.4	88.68	65.29	68.52	2.55	0.17
	0.45	84.28	71.95	73.65	3.00	0.22
	0.5	81.76	76.69	77.39	3.51	0.24
	0.55	76.73	80.52	80.00	3.94	0.29
	0.6	74.21	83.96	82.61	4.63	0.31
